# Synthetic anticoagulant octaparin targets mitochondrial cardiolipin-GSDMD axis to rescue redox homeostasis in sepsis

**DOI:** 10.1016/j.redox.2025.103877

**Published:** 2025-09-22

**Authors:** Shule Zhang, Cong Feng, Ning Yu, Rui Fang, Yingxin Zhang, Simeng Chen, Lijuan Cao, Jianfa Zhang

**Affiliations:** a State Key Laboratory of Natural Medicines, China Pharmaceutical University, Nanjing, 210009, China; b Center for Molecular Metabolism, Nanjing University of Science & Technology, Nanjing, 210094, China

**Keywords:** Sepsis, Redox homeostasis, Octaparin, Mitochondria, Pyroptosis, Cardiolipin

## Abstract

Sepsis, characterized by dysregulated immune responses and mitochondrial dysfunction, currently has few effective therapies that directly target these cellular mechanisms, and conventional heparin and related analogues provide inadequate immunomodulatory benefits. Here, we investigated the synthetic heparin analogue octaparin, which exhibits enhanced anticoagulant safety, for its potential to mitigate sepsis by targeting mitochondrial and redox pathways. Using murine models of lipopolysaccharide (LPS)-induced endotoxemia and *Salmonella typhimurium*-induced sepsis, along with in vitro studies performed using murine bone marrow-derived macrophages (BMDMs) and the human acute monocytic leukemia THP-1 cell line, we demonstrate that octaparin significantly improves survival and attenuates multi-organ (lung, liver, kidney) damage. Octaparin outperformed heparin, enoxaparin, and fondaparinux in suppressing systemic inflammation including TNF-α, IL-6, IL-1β and bacterial burden. Transcriptomic analysis revealed octaparin reprograms macrophage immunometabolism, suppressing pro-inflammatory pathways while enhancing phagocytosis. Crucially, octaparin inhibited both canonical and non-canonical inflammasome activation, reduced generation of the pyroptotic executor GSDMD-N-terminal fragment (GSDMD-NT), and specifically diminished mitochondrial localization of GSDMD-NT by downregulating key cardiolipin synthesis and transport genes. Furthermore, octaparin uniquely reversed LPS-induced mitochondrial dysfunction. This restoration was accompanied by improvements in mitochondrial quality and the reestablishment of redox homeostasis. Collectively, octaparin confers multifaceted protection in sepsis, positioning it as a promising redox-targeted therapeutic for sepsis.

## Highlights

• Octaparin outperforms heparin and related analogues in murine sepsis, improving survival, reducing multi-organ damage, inflammation and bacterial burden.
• Octaparin reverses mitochondrial dysfunction and reprograms macrophage immunometabolism to enhance phagocytosis.
• Octaparin suppresses GSDMD-NT generation and mitochondrial translocation by inhibiting inflammasomes and cardiolipin synthesis.

## 1 Introduction

Sepsis, a life-threatening syndrome driven by dysregulated host responses to infection, imposes a staggering global burden with an estimated 49 million cases and 11 million deaths annually [1]. Mortality exceeds 30 % overall and surpasses 50 % in severe cases despite advances in supportive care [[2], [3], [4], [5], [6]]. This persistent high fatality stems from the complex cellular pathogenesis of sepsis, which involves maladaptive crosstalk between hyperactivated immunity, coagulopathy, and mitochondrial dysfunction—a core hallmark characterized by impaired oxidative phosphorylation, excessive reactive oxygen species (ROS) generation, and disrupted organellar dynamics [[7], [8], [9], [10], [11], [12]]. Critically, damaged mitochondria release mitochondrial DNA (mtDNA) and damage-associated molecular patterns (DAMPs), activating inflammasomes such as Nod-like receptor pyrin domain-containing protein 3 (NLRP3) and driving gasdermin D (GSDMD)-mediated macrophage pyroptosis [[13], [14], [15], [16]]. This GSDMD-mediated pro-inflammatory cell death pathway liberates interleukin-1β (IL-1β), interleukin-18 (IL-18), and intracellular contents, establishing a self-amplifying cycle of organ injury and redox imbalance [17]. Recent evidence further indicates that mitochondrial damage extends beyond acute organ failure, with sepsis survivors exhibiting reprogramming of mitochondrial calcium uniporter (MCU) complexes in immune tissues, leading to persistent immunosuppression and chronic inflammation [18,19].

Current sepsis therapies remain inadequate due to their inability to address these cellular underpinnings [20]. Antimicrobials and immunomodulators show limited efficacy against heterogeneous immune responses [21,22], while metabolic interventions (e.g., thiamine supplementation to restore pyruvate dehydrogenase activity) face challenges in clinical translation [23,24]. Heparin and its analogues, including unfractionated heparin (UFH), low-molecular-weight heparins (LMWHs) like enoxaparin, and synthetic pentasaccharides such as fondaparinux, are widely used for thromboprophylaxis in sepsis [[25], [26], [27], [28]]. Clinical data suggest potential benefits such as shortened mechanical ventilation duration and intensive care unit (ICU) stays [29]. While UFH exhibits modest anti-inflammatory effects through anticoagulant-dependent mechanisms [[30], [31], [32]], all current heparin and related analogues share critical limitations: UFH carries risks of batch variability and heparin-induced thrombocytopenia (HIT); enoxaparin has reduced but non-negligible HIT risk; and fondaparinux, despite synthetic purity, shows negligible activity against mitochondrial dysfunction or pyroptotic pathways [[33], [34], [35]]. Critically, none mitigate mitochondrial GSDMD translocation, a process where the N-terminal pore-forming fragment of GSDMD (GSDMD-NT) binds cardiolipin on the outer mitochondrial membrane, exacerbating organelle damage and inflammasome activation [36].

To overcome these constraints, we developed octaparin, a synthetic heparin analogue with defined chemical structure and consistent factor Xa inhibitory activity. Our prior work confirmed that octaparin exhibits a superior safety profile with reduced bleeding risk relative to conventional heparin and its analogues, owing to its functional mimicry of heparin's anticoagulant activity [37]. Emerging evidence suggests that glycosaminoglycans play regulatory roles in immunometabolic processes. For example, they modulate stimulator of interferon genes (STING) -dependent inflammation through sulfation-pattern-specific interactions [[38], [39], [40], [41]], and endothelial heparan sulfate has been shown to mediate hepatic neutrophil trafficking and injury during *Staphylococcus aureus* sepsis [42]. Furthermore, heparan sulfate is increasingly recognized as a key modulator of mitochondrial dynamics and phospholipid metabolism [[43], [44], [45]]. Given these functions and the established involvement of cardiolipin, a mitochondria-specific phospholipid, in anchoring GSDMD-NT to initiate pyroptosis [46], we hypothesized that octaparin might disrupt mitochondrial pyroptotic signaling and thereby restore redox homeostasis.

In this study, we demonstrate that octaparin confers multifaceted protection against sepsis through interconnected mechanisms. It effectively suppresses systemic inflammation and enhances bacterial clearance, while concurrently inhibiting both canonical and non-canonical inflammasome activation pathways. Central to its action is the disruption of mitochondrial cardiolipin-GSDMD axis. Octaparin significantly reduces the mitochondrial translocation of the pore-forming GSDMD-NT fragment by downregulating two key regulators of cardiolipin metabolism: cardiolipin synthase (*Crls1*) and outer mitochondrial membrane phospholipid scramblase-3 (*Plscr3*). Concomitantly, it rescues sepsis-induced mitochondrial dysfunction through coordinated attenuation of reactive oxygen species (ROS) burst, restoration of membrane potential integrity, and reprogramming of metabolic pathways. These integrated actions, spanning immunomodulation, organelle quality control, and redox homeostasis, establish octaparin as a novel therapeutic agent targeting the critical intersection of mitochondrial stability and pyroptotic cell death in sepsis.

## 2 Materials and methods

### 2.1 Mice and treatment

Male C57BL/6J mice (6–8 weeks old) were purchased from the Experimental Animal Center of Nanjing University and used in this study. The mice were maintained in a 12-h light/dark cycle environment with temperatures ranging from 22 to 27 °C and humidity levels between 45 % and 55 %, with unrestricted access to food and water. To rigorously validate our hypothesis, we established two murine sepsis models: Lipopolysaccharide (LPS)-induced endotoxemia via intraperitoneal injection of LPS (25 mg/kg), with octaparin (10 or 20 mg/kg) administered intraperitoneally 30 min post-LPS; and *S. typhimurium*-induced infection via intraperitoneal challenge with 2 × 10^6^ CFU/20 g bacteria, followed by intraperitoneal octaparin (10 or 20 mg/kg) 2 h later. Mouse survival was monitored continuously throughout the experiment, or mice were sacrificed at 24 h after LPS and *S. typhimurium* injection to collect serum, as well as liver, lung, and kidney tissues for further analysis. All procedures followed the ARRIVE guidelines and were approved by the Institutional Animal Care and Use Committee of Nanjing University of Science and Technology (Approval No. IACUC-NJUST-2024-0058).

### 2.2 BMDM extraction

Bone Marrow-Derived Macrophages (BMDMs) were isolated from 6-week-old male C57BL/6 mice. Cells were maintained in complete Dulbecco's Modified Eagle Medium (DMEM, Gibco, 11995-065) supplemented with 50 ng/mL macrophage colony-stimulating factor (M-CSF, Novoprotein, CB34) at 37 °C under 5 % CO_2_. Following 7 days of culture, adherent cells were harvested as BMDMs for subsequent experiments.

### 2.3 Cell line

Human acute monocytic leukemia THP-1 cells (ATCC, TIB-202; male) were kindly provided by the Stem Cell Bank, Chinese Academy of Sciences (Shanghai, China), and authenticated via Short Tandem Repeat (STR) profiling. Prior to experiments, THP-1 cells were differentiated into macrophages by priming with 20 ng/mL phorbol 12-myristate 13-acetate (PMA, Sigma-Aldrich, P8139) for 48 h.

### 2.4 Microbe strains

*S. typhimurium* (ATCC, 14028) was procured from ATCC, while *S. typhimurium-GFP* was obtained from BeNa Culture Collection. Bacteria were revived and cultured on Luria-Bertani (LB, Fisher Bioreagents, BP1426-2) agar plates (2 % agar) at 37 °C overnight. A single colony was selected and amplified in LB broth at 37 °C overnight with shaking. Log-phase bacteria were harvested for infection experiments. For in vivo bacterial burden assessment, tissues were collected and homogenized in Phosphate-Buffered Saline (PBS). Diluted tissue homogenates were plated on LB-agar, and bacterial burdens in tissues were counted following 24 h culture.

### 2.5 Cell treatment

To induce canonical inflammasome-mediated pyroptosis, BMDMs were primed with 1 μg/mL LPS (Sigma-Aldrich, L3024) for 2 h, and then stimulated with 5 mM Adenosine Triphosphate (ATP, TargetMol, T1352) or 10 μM nigericin (MCE, HY-127019) for 2 h. To induce non-canonical inflammasome-mediated pyroptosis, BMDMs were either transfected with LPS (5 μg/mL) using 0.3 % (v/v) Fugene HD (Promega, E231A) for 24 h, or treated with LPS (1 μg/mL) plus High mobility group protein B1 (HMGB1, MCE, HY-P73104) (100 ng/mL) for 24 h. For the *S. typhimurium* infection assay, bacteria were cultured to mid-log phase, washed with PBS, and added to cells at the indicated Multiplicity of Infection (MOI). Next, 100 μg/mL gentamicin (Thermo Fisher, 15750-060) was added to the cultures 2 h after infection to kill extracellular bacteria. Fresh media containing 20 μg/mL gentamicin was then added back to the cells for the duration of infection. To assess the relative efficacy of octaparin, heparin and its analogues in mitigating sepsis-associated inflammation, BMDMs were stimulated with either LPS (1 μg/mL) or *S. typhimurium* (MOI = 10) for 24 h in the presence of octaparin (synthetic heparin analogue, in-house synthesized; purity ≥95 % confirmed by HPLC, 5 μg/mL), heparin (MCE, HY-17567, 5 μg/mL), enoxaparin (MCE, HY-109509, 5 μg/mL), and fondaparinux (MCE, HY-B0597, 5 μg/mL).

### 2.6 Flow cytometric analysis

The differentiation of THP-1 cells into macrophages was verified by flow cytometry with a PE-conjugated anti-human CD68 antibody (BioLegend, 333816, 1:200). Cells were acquired on a FACSCelesta flow cytometer, and data were analyzed using FlowJo-V10 software.

### 2.7 Enzyme-Linked Immunosorbent Assay (ELISA) and clinical chemistry analysis

Serum samples were prepared by centrifuging coagulated blood twice at 500×*g* 10 min and 4 °C. Cell culture supernatants were obtained by centrifuging the medium at 300×*g* for 10 min, and these supernatants were collected for subsequent analysis. All samples were processed following the manufacturers' protocols. The levels of human IL-1α (ABclonal, RK00031), mouse IL-1α (ABclonal, RK04876), human IL-1β (ABclonal, RK00001), mouse IL-1β (ABclonal, RK04878), human Tumor Necrosis Factor-alpha (TNF-α, ABclonal, RK05051), mouse TNF-α (ABclonal, RK04875), human IL-6 (ABclonal, RK04769) and mouse IL-6 (ABclonal, RK04845) were determined by ELISA kits. Serum Lactate Dehydrogenase (LDH, A020-2-2), Alanine Aminotransferase (ALT, C009-2-1), Aspartate Aminotransferase (AST, C010-2-1), creatinine (Cr, C011-2-1) and blood urea nitrogen (BUN, C013-2-1) were analyzed by colorimetry using respective kits from Nanjing Jiancheng Bioengineering Institute according to the manufacturer's instructions.

### 2.8 Western blot

Cells were seeded in 6-well plates and treated with experimental compounds for 24 h. Whole-cell lysates were prepared using radioimmunoprecipitation assay buffer (RIPA, Beyotime Biotechnology, P0013B), resolved by SDS-PAGE, and transferred to Polyvinylidene Fluoride (PVDF) membranes. Membranes were blocked with 5 % nonfat milk in Tris-Buffered Saline with 0.1 % Tween-20 (TBST) for 1 h at room temperature, incubated with primary antibodies overnight at 4 °C, washed five times with TBST, probed with secondary antibodies (goat anti-rabbit IgG, Abcam, ab6721, 1:5000; goat anti-mouse IgG, Abcam, ab6789, 1:5000) for 1 h at room temperature, and visualized using enhanced chemiluminescence (ECL, Thermo Fisher, 34578) substrate with a chemiluminescence imaging system. The primary antibodies used were: Caspase-1 (ABclonal, A21085, 1:1000), Caspase-11 (abcam, ab180673, 1:1000), GSDMD (abcam, ab209845, 1:1000), CRLS1 (Proteintech, 51055-1-AP, 1:500), PLSCR3 (Huabio, HA722577, 1:1000), Actb (beta-actin) (ABclonal, AC026, 1:5000), Voltage-Dependent Anion Channel (VDAC) (abcam, ab14734, 1:2000) and α-tubulin (ABclonal, A6830, 1:2000).

### 2.9 RNA-seq analysis

BMDMs were seeded in 60-mm dishes and stimulated with LPS (1 μg/mL) alone or in combination with octaparin (5 μg/mL) for 24 h. Total RNA was extracted using TRIzol reagent (Thermo Fisher, 15596018). RNA quality was assessed via the Bioanalyzer 2100 system with the RNA 6000 Nano LabChip Kit (Agilent, CA, USA, 5067-1511); only samples with an RNA integrity number (RIN) ≥7.0 were advanced for library preparation. RNA-seq was performed by Majorbio Bio-Pharm Technology Co., Ltd. (Shanghai, China) on the Illumina NovaSeq platform. Differentially expressed genes (DEGs) were identified using a false discovery rate (FDR) < 0.05 and absolute fold change ≥2. Gene Set Enrichment Analysis (GSEA) was conducted to assess pathway-level enrichment, complemented by functional enrichment analysis via Gene Ontology (GO) and Kyoto Encyclopedia of Genes and Genomes (KEGG) databases.

### 2.10 Hematoxylin-eosin (H&E) staining

Mice were sacrificed at 24 h after injection with either LPS or *S. typhimurium* to collect liver, lung, and kidney tissues. These tissues were fixed overnight in 4 % paraformaldehyde, followed by dehydration, paraffin embedding, sectioning, and staining with hematoxylin and eosin (H&E). The relevant pathological sections were prepared by Nanjing Youmeng Biotechnology Co., Ltd. Sections were visualized and imaged using light microscopy.

### 2.11 RNA isolation and RT-qPCR analysis

Total RNA was extracted from BMDMs using TRIzol reagent (Thermo Fisher, 15596018) per the manufacturer's protocol. RNA purity and concentration were assessed via NanoDrop spectrophotometer (Agilent Technologies). Single-stranded cDNA was synthesized using HiScript II QRT SuperMix for qPCR (Vazyme, R223-01). RT-qPCR was performed using ChamQ SYBR Color qPCR Master Mix (Vazyme, Q431-02) on a real-time PCR system. Relative gene expression was calculated by the 2^−ΔΔCT^ method, normalizing to *Actb (beta-actin)*. Primers were synthesized by Sangon Biotech (China, Shanghai). The primer sequences used were as follows (5′–3′):

*Crls1*: forward, GCCAGCTCGTATGAAAATCCA; reverse, GCAAAAACACCTAGTGCAACATT.

*Plscr3*: forward, CCTGCTCCTTTCGTGCCATT; reverse, CCACTCGTTCAGCCTTCTGAT.

*Actb*: forward, GTGACGTTGACATCCGTAAAGA; reverse, GCCGGACTCATCGTACTCC.

### 2.12 Cell death assays

Cell viability was determined by quantifying ATP levels using the CellCounting-Lite® 2.0 Luminescent Cell Viability Assay (Vazyme, DD1101-02). Cell death was assessed with the LDH Cytotoxicity Assay kit (Beyotime Biotechnology, C0017) following the manufacturer's instructions. For visualization of morphological alterations, BMDMs were seeded onto glass-bottom dishes and treated as specified. After staining with 200 ng/mL propidium iodide (MCE, HY-D0815) for 15 min at room temperature (RT), fluorescence images were acquired using an LSM800 confocal microscope (Zeiss).

### 2.13 Mitochondrial isolation

BMDMs were washed with PBS and gently scraped for collection. Mitochondria were isolated using the Mitochondria Isolation Kit (MCE, HY-K1060) following the manufacturer's protocol. The efficiency of mitochondrial isolation was verified by Western blotting.

### 2.14 Cell mitochondrial function assays

To visualize mitochondrial morphology in BMDMs, cells were stained with 200 nM MitoTracker Deep Red (Thermo Fisher, M22426) at 37°C for 30 min. Reactive oxygen species (ROS), mitochondrial superoxide, and mitochondrial transmembrane potential were assessed using H2DCFDA (MCE, HY-D0940), MitoSOX Red (Thermo Fisher, M36008), and TMRM (MCE, HY-D0984A) staining, respectively, following the manufacturers’ instructions.

### 2.15 Seahorse metabolic analysis

BMDMs (1 × 10^5^ cells/well) were seeded in a Seahorse 96-well plate with DMEM supplemented with 10 % fetal bovine serum (FBS, Gibco, 10437-028). Cells were treated with LPS (1 μg/mL) in the presence or absence of octaparin (5 μg/mL) for 24 h. Following treatment, cells were washed twice and incubated in Seahorse Assay Medium containing 25 mM glucose and 2 mM glutamine at 37 °C for 45 min. The oxygen consumption rate (OCR) was measured using the Seahorse XF Cell Mito Stress Test kit (Agilent, 103015-100) under basal conditions and after sequential injection of 1.5 μM oligomycin, 1.5 μM FCCP, and 0.5 μM rotenone plus 0.5 μM antimycin A using a Seahorse XFe96 Extracellular Flux Analyzer (Agilent).

### 2.16 Phagocytosis activity assay

For phagocytosis activity assessment using neutral red, BMDMs were treated with octaparin for 24 h. Subsequently, cells were incubated with 0.05 % neutral red (in PBS) for 1 h. The neutral red solution was then discarded, and the cells were washed twice with PBS. A dissolution solution containing 50 % ethanol, 1 % acetic acid, and 49 % H_2_O was added, and the absorbance at 540 nm was measured using a Synergy H1 multi-mode reader (BioTek, Winooski, VT, USA). For phagocytosis activity assessment using *S. typhimurium-GFP*, BMDMs were subjected to two distinct treatment protocols: in the first protocol, cells were treated with octaparin for 24 h prior to a 2 h incubation with *S. typhimurium-GFP* at an MOI of 10, followed by phagocytic activity quantification; in the second protocol, BMDMs were first incubated with *S. typhimurium-GFP* at an MOI of 10 for 1 h, then stimulated with octaparin at the indicated doses for an additional 24 h before phagocytic activity was quantified. Extracellular bacteria were then thoroughly washed away with PBS twice, and the fluorescence intensity at Ex_488_ nm/Em_525_ nm was recorded using a Synergy H1 multi-mode reader (BioTek, Winooski, VT, USA). For assessment of extracellular bacterial clearance, BMDMs were subjected to two treatment conditions: in the first, BMDMs pre-stimulated with octaparin at the indicated doses for 24 h were infected with *S. typhimurium-GFP* at an MOI of 10 for 6 h; in the second, BMDMs were first incubated with *S. typhimurium-GFP* at an MOI of 10 for 1 h, followed by stimulation with the indicated doses of octaparin for an additional 6 h. Extracellular bacteria were visualized using an LSM800 confocal microscope (Zeiss).

### 2.17 Immunofluorescence

Processing of tissue sections or cells was performed sequentially at room temperature: fixation in 4 % paraformaldehyde for 20 min, permeabilization with 0.1 % Triton X-100 (Sigma-Aldrich, T8787) for 15 min, and blocking with 5 % BSA (Beyotime Biotechnology, ST025) for 30 min. Samples were then incubated overnight at 4 °C with primary antibodies. The following day, tissues and cells were stained with fluorochrome-conjugated secondary antibodies for 1 h in the dark, followed by nuclear counterstaining with 4′,6-Diamidino-2-Phenylindole (DAPI, Sigma-Aldrich, MBD0015) for 10 min. Confocal imaging was performed using an LSM800 confocal microscope (Zeiss). The primary antibodies used were: F4/80 (ABclonal, A23788, 1:200), Cleaved Caspase-3 (CST, 9661, 1:100), Cleaved GSDMD (CST, 10137, 1:100).

### 2.18 Statistical analysis

Statistical analyses were performed using GraphPad Prism 8.4.3. All error bars represent the mean ± SEM, and the number of independent experiments (n) for each figure is specified in the legend. For comparisons between two groups, a two-sided Student's t-test was applied. For multiple group comparisons, one-way or two-way ANOVA was used, with post hoc Bonferroni correction for pairwise within-group comparisons. Survival data were analyzed using the log-rank test and Kaplan-Meier curves. A p-value <0.05 was considered statistically significant across all analyses.

## 3 Results

### 3.1 Octaparin improves survival in sepsis by attenuating inflammatory-driven organ failure

**Fig. 1:**
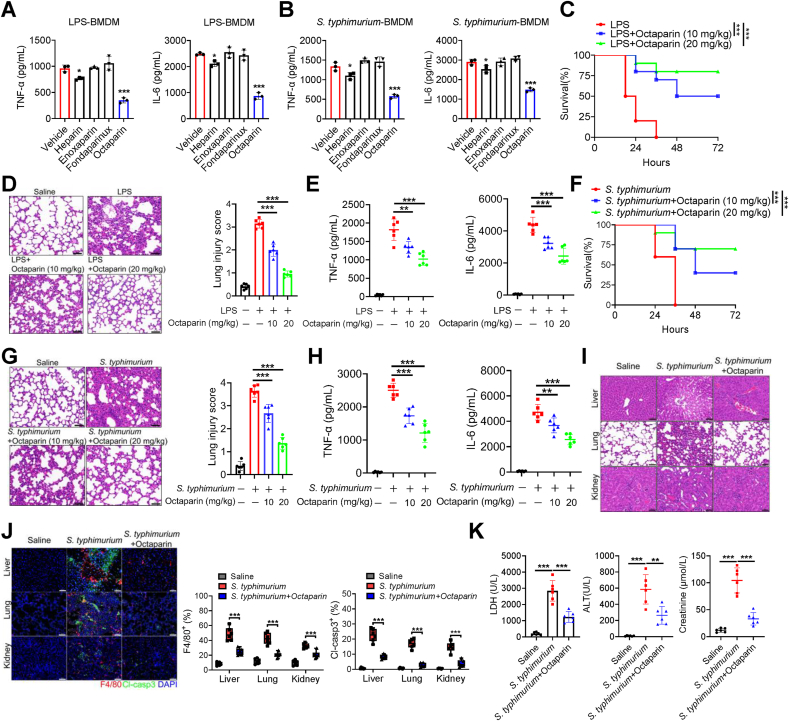
Octaparin attenuates systemic inflammation and organ failure to improve survival in sepsis. (A) ELISA analysis of TNF-α and IL-6 in culture supernatants from BMDMs stimulated with LPS (1 μg/mL) in the presence or absence of octaparin (5 μg/mL), heparin (5 μg/mL), enoxaparin (5 μg/mL) and fondaparinux (5 μg/mL) for 24 h. (B) ELISA analysis of TNF-α and IL-6 in culture supernatants from BMDMs stimulated with *S. typhimurium* (MOI = 10) in the presence or absence of octaparin (5 μg/mL), heparin (5 μg/mL), enoxaparin (5 μg/mL) and fondaparinux (5 μg/mL) for 24 h. (C–E) Kaplan-Meier survival curves (C), histopathological images of lung tissues with quantitative assessment of lung injury via scoring of histopathological features (D), and serum concentrations of TNF-α and IL-6 (E) from mice at 24 h after intraperitoneal injection of LPS (25 mg/kg) or saline. Octaparin (10 or 20 mg/kg) was administered intraperitoneally 30 min after LPS injection. Scale bar represents 50 μm. (F–H) Kaplan-Meier survival curves (F), histopathological images of lung tissues with quantitative assessment of lung injury via scoring of histopathological features (G), and serum concentrations of TNF-α and IL-6 (H) from mice at 24 h after intraperitoneal injection of *S. typhimurium* (2 × 10^6^ CFU/20 g) or saline. Octaparin (10 or 20 mg/kg) was administered intraperitoneally 2 h after *S. typhimurium* injection. Scale bar represents 50 μm. (I–K) Mice received intraperitoneal injections of *S. typhimurium* (2x106 CFU/20 g) or sterile saline. Octaparin (20 mg/kg) was administered intraperitoneally 2 h following *S. typhimurium* challenge. Mice were sacrificed 48 h later for various analyses. Representative H&E-stained sections of liver, lung, and kidney tissues. Scale bars, 50 μm (I). Immunofluorescence staining for F4/80 and Cl-casp3 in liver, lung, and kidney tissues. Percentage of F4/80^+^ and Cl-casp3^+^ cells in liver, lung, and kidney tissues. Scale bar, 50 μm (J). Serum levels of LDH, ALT and creatinine (K). The graphs are shown as individual data points along with mean ± SEM. ∗p < 0.05; ∗∗p < 0.01; ∗∗∗p < 0.001. Statistical analyses by one-way ANOVA test and log-rank test for survival.

To evaluate the comparative efficacy of octaparin, heparin, and related analogues in reducing sepsis-associated inflammation, we began by analyzing their structural characteristics, such as sulfation patterns and molecular weights (Supplemental Fig. 1A). In both LPS- and *S. typhimurium*-stimulated BMDMs, octaparin strongly inhibited TNF-α and IL-6 release, while heparin showed only modest suppression, and enoxaparin or fondaparinux had negligible effects (Fig. 1A and B). To confirm the cross-species efficacy of octaparin, we used the differentiation of THP-1 cells into macrophages by flow cytometry with a PE-conjugated anti-human CD68 antibody (Supplemental Fig. 2). Octaparin exhibited comparable anti-inflammatory potency in human THP-1 macrophages challenged with either LPS or *S. typhimurium* (Supplemental Fig. 1B and C). Octaparin also conferred significant protection in murine sepsis models. In LPS-challenged mice, it improved survival in a dose-dependent manner (Fig. 1C), attenuated lung injury (Fig. 1D), and reduced systemic levels of TNF-α, IL-6, IL-1α, and IL-1β (Fig. 1E; Supplemental Fig. 1D). Comparable benefits were observed in *S. typhimurium*-infected mice, including enhanced survival (Fig. 1F), reduced pulmonary damage (Fig. 1G), and suppressed cytokine release (Fig. 1H; Supplemental Fig. 1E).

Furthermore, octaparin mitigated sepsis-induced multiple organ failure. In *S. typhimurium*-infected mice, it reduced inflammatory infiltration and preserved tissue architecture in the liver, lung, and kidney (Fig. 1I). Immunofluorescence analysis revealed decreased F4/80^+^ macrophage accumulation and cleaved caspase-3^+^ cells (Fig. 1J), indicating attenuated inflammation and cell death. Consistently, octaparin treatment lowered serum markers of organ damage, including LDH, ALT, AST, creatinine, and BUN (Fig. 1K; Supplemental Fig. 3A and B). Collectively, these results indicate that octaparin alleviates septic lethality and multi-organ dysfunction by suppressing inflammatory responses, limiting immune cell infiltration, and reducing cellular damage.

### 3.2 Transcriptional reprogramming of macrophage immunometabolism

**Fig. 2:**
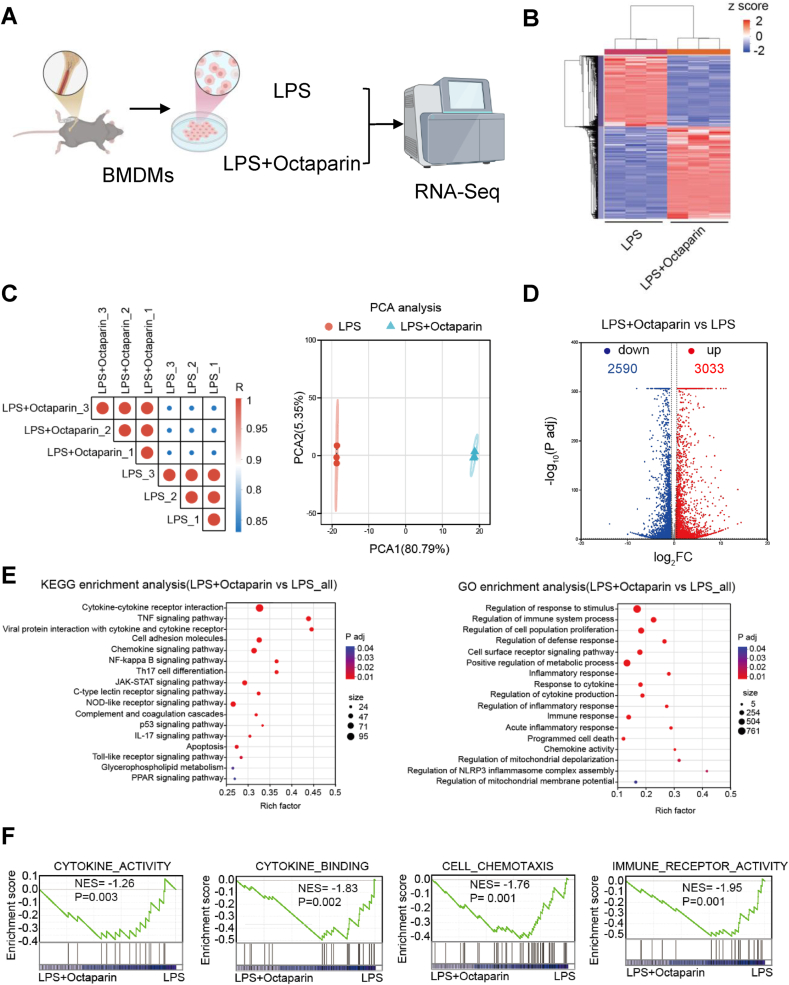
Octaparin regulates the inflammation-related metabolic reprogramming of macrophages in sepsis. (A) Schematic overview of the experimental design of RNA-seq. BMDMs were stimulated with LPS alone (1 μg/ml) or LPS combined with octaparin (5 μg/ml) for 24 h. (B) Heatmap of differentially expressed genes in LPS + octaparin vs. LPS treatment groups (n = 3). (C) Pearson correlation between samples and their PCA plot. (D) Volcano plot of differentially expressed genes. (E) KEGG and GO enrichment analysis of differentially expressed genes in BMDMs following treatment with LPS plus octaparin or LPS alone. (F) GSEA results for gene-sets related to cytokine activity, cytokine binding, cell chemotaxis, and immune receptor activity in LPS + octaparin vs. LPS treatment groups (n = 3).

To dissect the immunomodulatory mechanisms underlying organ protection, we next sought to dissect the underlying transcriptional basis using RNA sequencing (RNA-seq). Specifically, we profiled LPS-stimulated BMDMs with or without octaparin treatment to identify key molecular pathways involved (Fig. 2A). Octaparin treatment induced distinct shifts in the transcriptional profiles of LPS-activated BMDMs (Fig. 2B). PCA analysis confirmed robust reproducibility within groups and clear separation between treatment conditions, validating the reliability of observed gene-expression differences (Fig. 2C). Transcriptomic analysis identified 5623 significantly differentially expressed genes in octaparin-treated cells (Fig. 2D). KEGG and GO enrichment analyses of these genes highlighted enrichment in pathways central to sepsis pathogenesis, including cytokine-cytokine receptor interaction, TNF signaling, and inflammatory response, among others, consistent with a role for octaparin in reshaping macrophage immunometabolic responses (Fig. 2E). Focused Gene Set Enrichment Analysis (GSEA) further revealed that octaparin downregulated gene sets associated with cytokine activity, cytokine binding, cell chemotaxis, and immune receptor signaling (Fig. 2F). These findings not only align with our above observation in Fig. 1 that octaparin inhibits pro-inflammatory cytokine production in BMDMs but also provide transcriptional evidence that such inhibition stems from broad suppression of pro-inflammatory transcriptional programs in macrophages. Collectively, these data establish a transcriptional basis for octaparin's ability to regulate macrophage metabolic reprogramming and subsequent pro-inflammatory phenotypes, thereby underpinning its immunomodulatory potential in sepsis.

### 3.3 Phagocytosis enhancement without pyroptosis induction

**Fig. 3:**
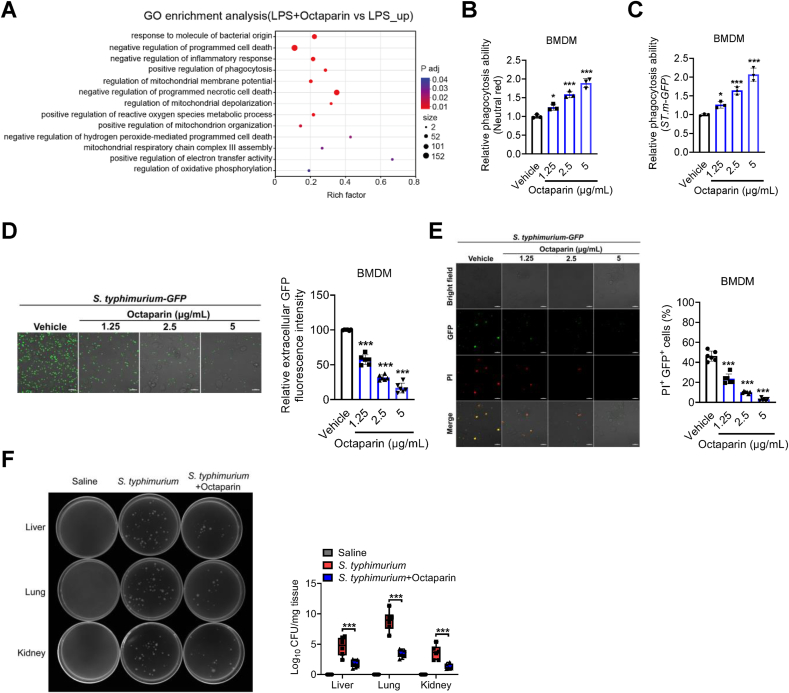
Octaparin potently enhances the phagocytic capacity of BMDMs. (A) GO enrichment analysis of upregulated genes in BMDMs following treatment with LPS plus octaparin versus LPS alone. (B) The relative phagocytic activity of BMDMs stimulated with indicated doses of octaparin for 24 h was assessed via neutral red uptake. (C) The relative phagocytic activity of BMDMs stimulated with indicated doses of octaparin for 24 h was assessed via *S. typhimurium-GFP* uptake. (D) BMDMs were stimulated with indicated doses of octaparin for 24 h, followed by incubation with *S. typhimurium-GFP* at a MOI of 10 for 6 h. Representative images are shown. Bacterial clearance capacity was evaluated by calculating the percentage decrease in extracellular fluorescence. Scale bars, 20 μm. (E) BMDMs were stimulated with indicated doses of octaparin for 24 h, followed by incubation with *S. typhimurium-GFP* at a MOI of 5 for 24 h. Representative confocal microscopy images show PI ^+^ GFP ^+^ cells. Scale bar, 20 μm. (F) Mice received intraperitoneal injections of *S. typhimurium* (2 × 10^6^ CFU/20 g) or sterile saline. Octaparin (20 mg/kg) was given intraperitoneally 2 h post *S. typhimurium* challenge, and mice were sacrificed 48 h later for analyses. Bacterial loads in the liver, lung, and kidney were determined. The graphs are shown as individual data points along with mean ± SEM. ∗p < 0.05; ∗∗p < 0.01; ∗∗∗p < 0.001. Statistical analyses by one-way ANOVA test.

To dissect the immunomodulatory mechanisms of octaparin in BMDMs, we performed Gene Ontology (GO) enrichment analysis on genes upregulated in the LPS + octaparin group relative to the LPS-only group. The analysis revealed significant enrichment of pathways including negative regulation of programmed cell death, positive regulation of phagocytosis, and modulation of mitochondrial dynamics (Fig. 3A). These findings suggest octaparin may orchestrate this tripartite relationship to enhance phagocytosis while limiting mitochondrial-mediated pyroptosis and excessive inflammatory responses in BMDMs. Thus, we first set out to investigate whether octaparin could enhance phagocytic activity and bacterial clearance capacity. Surprisingly, octaparin significantly increased the phagocytic activity of BMDMs in a dose-dependent manner, with relative phagocytic capacity validated by neutral red uptake and *S. typhimurium-GFP* phagocytosis assays (Fig. 3B and C). Meanwhile, confocal microscopy images revealed a reduction in extracellular *S. typhimurium-GFP* fluorescence intensity following octaparin treatment, indicating that octaparin enhances the bacterial clearance capacity of BMDMs (Fig. 3D). Subsequently, we assessed the impact of octaparin on pyroptosis triggered by *S. typhimurium-GFP* phagocytosis in BMDMs. Notably, octaparin enhances BMDM phagocytosis of bacteria while concomitantly attenuating pyroptosis elicited by the phagocytic process (Fig. 3E). Notably, when assayed using a treatment regimen that matches our in vivo protocol, the octaparin-mediated enhancement of phagocytic function in BMDMs is fully recapitulated (Supplemental Fig. 4). This consistency aligns with our original in vitro observations and highlights the robustness of octaparin's pro-phagocytic activity in BMDMs, which is not dependent on the timing of its administration relative to inflammatory stimulation. To validate the in vivo relevance, we evaluated the impact of octaparin on bacterial clearance in vital organs. In *S. typhimurium*-infected mice, octaparin treatment potently decreased bacterial load in the liver, lung, and kidney tissues (Fig. 3F). Collectively, these results underscore octaparin's capacity to orchestrate the tripartite relationship between phagocytosis, mitochondrial dynamics, and programmed cell death, thereby balancing antimicrobial defense and inflammatory homeostasis.

### 3.4 Inhibition of canonical and non-canonical inflammasomes

**Fig. 4:**
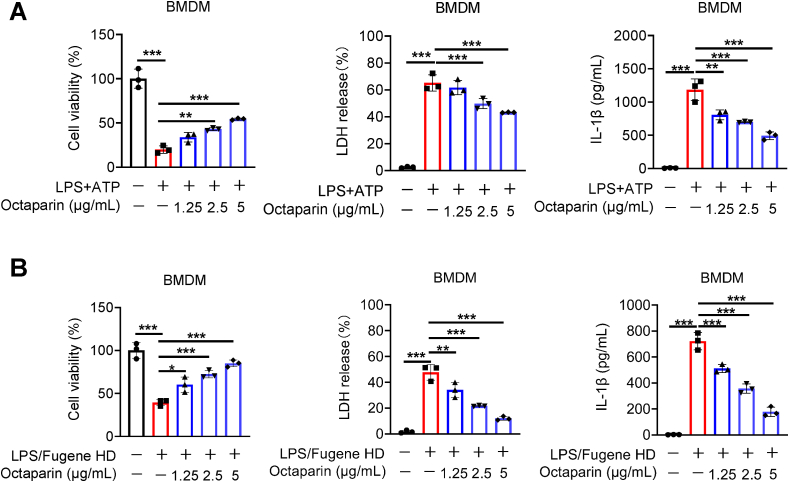
Octaparin inhibits canonical and non-canonical inflammasome activation in BMDMs. (A) Cell viability, LDH release, and IL-1β secretion in BMDMs primed with LPS (1 μg/mL) for 2 h, followed by ATP (5 mM) stimulation for 2 h in the presence or absence of indicated octaparin doses. (B) Cell viability, LDH release, and IL-1β secretion in BMDMs transfected with LPS (5 μg/mL) via Fugene HD for 24 h in the presence or absence of indicated octaparin doses. The graphs are shown as individual data points along with mean ± SEM. ∗p < 0.05; ∗∗p < 0.01; ∗∗∗p < 0.001. Statistical analyses by one-way ANOVA test.

To define how octaparin limits pyroptosis during bacterial clearance, we next examined whether octaparin regulates canonical and non-canonical inflammasome activation in BMDMs. Inflammasome activation is a pivotal event in innate immunity, orchestrating host defense against pathogens and the initiation of inflammatory responses [47]. Within macrophages, both canonical and non-canonical inflammasome pathways mediate the detection of danger signals and initiate cascades driving pro-inflammatory cytokine maturation and release [48]. First, we investigated whether octaparin exerts inhibitory effects on canonical inflammasome activation. As expected, octaparin dose-dependently inhibited LPS + ATP-induced canonical inflammasome activation in BMDMs, evidenced by improved cell viability concurrent with reduced LDH release and IL-1β secretion (Fig. 4A). This inhibitory effect was similarly observed in LPS/nigericin-induced canonical inflammasome activation (Supplemental Fig. 5A). Next, we explored whether octaparin inhibits non-canonical inflammasome activation, which is defined by direct recognition of intracellular LPS. Octaparin dose-dependently suppressed LPS transfection-induced non-canonical inflammasome activation, as shown by increased cell viability and reduced LDH release and IL-1β secretion (Fig. 4B). Previous studies have shown that HMGB1 enables extracellular LPS to induce non-canonical inflammasome activation in macrophages, which is critical for the activation of coagulation cascades during endotoxemia [49,50]. Strikingly, octaparin dose-dependently suppressed the cytotoxicity and pro-inflammatory response triggered by HMGB1-LPS complexation, as evidenced by reduced loss of cell viability, decreased LDH release, and diminished IL-1β secretion in BMDMs (Supplemental Fig. 5B). Taken together, these data demonstrate that octaparin potently inhibits both canonical and non-canonical inflammasome activation in BMDMs.

### 3.5 Cardiolipin-mediated blockade of mitochondrial GSDMD translocation

**Fig. 5:**
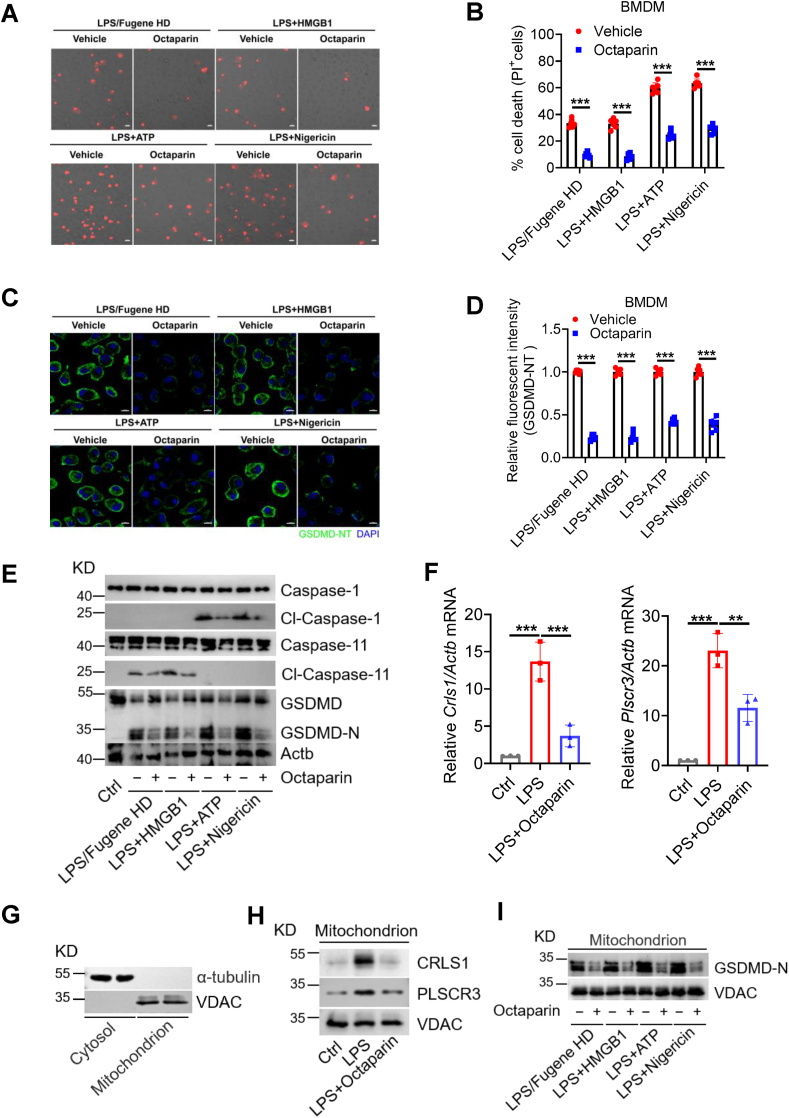
**Octaparin diminishes GSDMD-NT mitochondrial localization in pyroptotic BMDMs.** BMDMs were treated as follows: primed with LPS (1 μg/mL) for 2 h, followed by stimulation with ATP (5 mM) or nigericin (10 μM) for 2 h; transfected with LPS (5 μg/mL) via Fugene HD for 24 h; or treated with LPS (1 μg/mL) + HMGB1 (100 ng/mL) for 24 h. All conditions included the presence or absence of octaparin (5 μg/mL). (A) Representative confocal microscopy images show PI-positive cells; scale bar, 20 μm. (B) Quantification of PI-positive cells was derived from (A). (C) Representative immunofluorescence images of GSDMD-NT in BMDMs stimulated as described. Scale bar, 10 μm. (D) Quantification of Relative fluorescent intensity (GSDMD-NT) was derived from (C). (E) Immunoblots of caspase-1/11 and GSDMD in BMDMs stimulated as described. (F) Relative mRNA levels of *Crls1* and *Plscr3* in BMDMs treated with LPS (1 μg/mL) for 24 h, with or without octaparin (5 μg/mL). (G) BMDMs were harvested for subcellular fractionation to isolate mitochondria, with isolation efficiency verified by immunoblotting. (H) Immunoblots of CRLS1 and PLSCR3 in mitochondrial fractions from BMDMs treated with LPS (1 μg/mL) for 24 h, in the absence or presence of octaparin (5 μg/mL). (I) Immunoblots of GSDMD-NT in mitochondrial fractions from BMDMs stimulated as described. The graphs are shown as individual data points along with mean ± SEM. ∗p < 0.05; ∗∗p < 0.01; ∗∗∗p < 0.001. Statistical analyses by one-way ANOVA or two-way ANOVA test.

To further investigate the specific mechanism by which octaparin inhibits pyroptosis, we next evaluated its effects on pyroptotic morphology and GSDMD activation in BMDMs. Numerous previous studies have shown that mitochondrial damage releases mtDAMPs that activate inflammasomes, boosting inflammation [51]. Conversely, pyroptotic triggers induce mitochondrial depolarization, ROS, and permeability transition, forming a feedforward loop amplifying cell death and inflammation [52,53]. Octaparin inhibits plasma membrane rupture and significantly reduces the percentage of propidium iodide (PI)-positive pyroptotic cells in BMDMs pyroptosis models induced by LPS/Fugene HD, LPS + HMGB1, LPS + ATP, and LPS + Nigericin (Fig. 5A and B). Meanwhile, immunofluorescence results also demonstrate that octaparin suppresses the activation and plasma-membrane-associated oligomerization of GSDMD-NT (Fig. 5C and D). Moreover, Western blot analysis revealed that octaparin suppressed GSDMD cleavage and the generation of the GSDMD-NT in both canonical and noncanonical inflammasome pathways, accompanied by reduced activation of caspase-1 and caspase-11 (Fig. 5E). This suggests that octaparin may inhibit pyroptosis by affecting the upstream signaling of inflammasome activation. Previous research has indicated that the permeabilization of mitochondrial inner and outer membranes mediated by gasdermin D expedites and intensifies pyroptosis [54]. We subsequently investigated whether octaparin influences the mitochondrial localization of GSDMD-NT. Given that the binding of GSDMD-NT to cardiolipin on the outer mitochondrial membrane is a prerequisite for mitochondrial damage [36], and combined with our aforementioned transcriptome analysis showing that octaparin regulates glycerophospholipid metabolism, we speculate that octaparin affects cardiolipin metabolism. Cardiolipin is synthesized by cardiolipin synthase (CRLS1) and translocated to the outer mitochondrial membrane by phospholipid scramblase-3 (PLSCR3) [55]. Consistent with our speculation, octaparin treatment significantly reduced the relative mRNA levels of *Crls1* and *Plscr3* compared to the LPS-treated group (Fig. 5F). Furthermore, we isolated the mitochondrial fractions from BMDMs, and the isolation efficiency was verified by Western-blot analysis (Fig. 5G). As expected, octaparin significantly reduced the relative protein expression of CRLS1 and PLSCR3 in mitochondria compared to the LPS-treated, and diminished the mitochondrial localization of GSDMD-NT during pyroptosis (Fig. 5H and I). Taken together, these data suggest that octaparin inhibits inflammasome activation and pyroptosis by reducing the synthesis and transport of cardiolipin, thereby preventing GSDMD-NT-induced mitochondrial damage.

### 3.6 Restoration of mitochondrial architecture and redox function

**Fig. 6:**
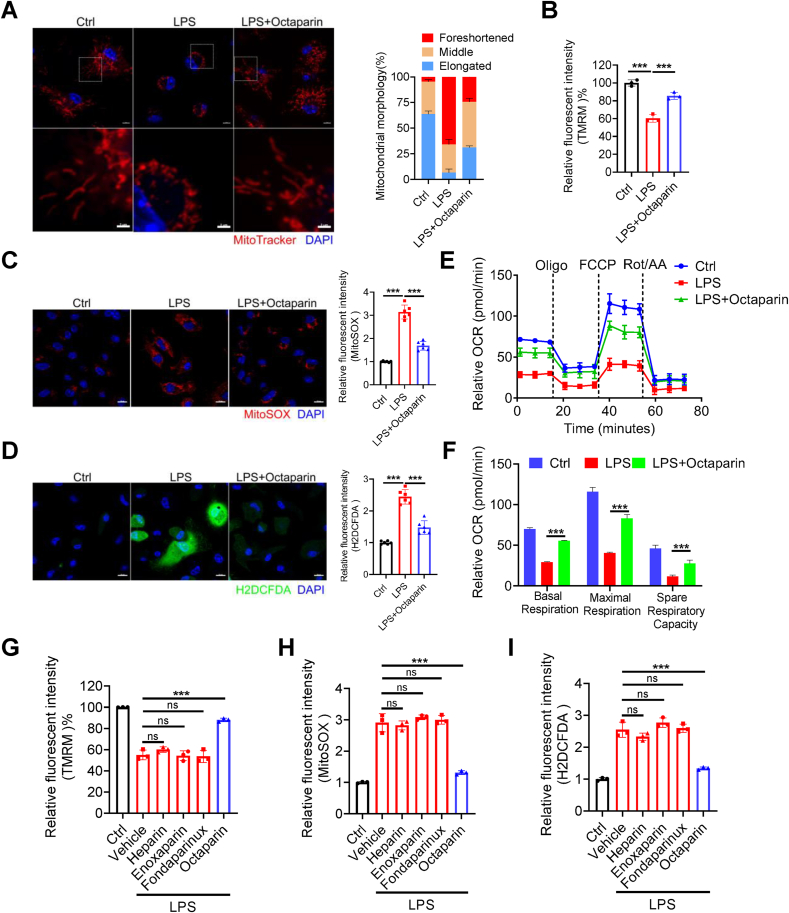
Octaparin inhibits LPS-mediated mitochondrial quality imbalance and restoration of redox homeostasis. (A) Representative confocal microscopy images of mitochondria in BMDMs treated with LPS (1 μg/mL) for 24 h, with or without octaparin (5 μg/mL). Mitochondrial morphology was categorized as foreshortened, middle, and elongated and subsequently quantified. Scale bar, 10 μm. (B) Relative fluorescence intensity of TMRM in BMDMs treated with LPS (1 μg/mL) for 24 h, with or without octaparin (5 μg/mL). (C) Representative confocal microscopy images of MitoSOX in BMDMs treated with LPS (1 μg/mL) for 24 h, with or without octaparin (5 μg/mL). Relative fluorescence intensity of MitoSOX in BMDMs. Scale bar, 10 μm. (D) Representative confocal microscopy images of reactive oxygen species (ROS) production in BMDMs treated with LPS (1 μg/mL) for 24 h, with or without octaparin (5 μg/mL). Relative fluorescence intensity of H2DCFDA in BMDMs. Scale bar, 10 μm. (E) Seahorse analysis of OCR in BMDMs treated with LPS (1 μg/mL) for 24 h, with or without octaparin (5 μg/mL). (F) Basal respiration, maximal respiration, and spare respiratory capacity were quantified from (E). (G–I) Relative fluorescence intensity of TMRM (G), MitoSOX (H) and H2DCFDA (I) in BMDMs treated with LPS (1 μg/mL) for 24 h in the presence or absence of octaparin (5 μg/mL), heparin (5 μg/mL), enoxaparin (5 μg/mL) and fondaparinux (5 μg/mL). The graphs are shown as individual data points along with mean ± SEM. ∗p < 0.05; ∗∗p < 0.01; ∗∗∗p < 0.001. Statistical analyses by two-way ANOVA or Student's t-test.

Given that mitochondrial quality imbalance and disrupted redox homeostasis are closely linked to pyroptosis [53], mitochondrial dysfunction drives cellular damage, particularly in inflammation during sepsis pathogenesis [56]. We next sought to further investigate the role of octaparin in maintaining mitochondrial homeostasis by exploring its impact on LPS-induced mitochondrial dysfunction in BMDMs. First, we investigated the specific effects of different treatments on mitochondrial morphology. Confocal microscopy images revealed that LPS-stimulated BMDMs exhibited characteristic mitochondrial fragmentation with a higher proportion of foreshortened mitochondria. In contrast, octaparin significantly ameliorated this fragmentation, as evidenced by increased mitochondrial fusion and a higher proportion of elongated mitochondria, indicating a marked improvement in mitochondrial morphology (Fig. 6A). Mitochondria-derived superoxide (MitoSOX intensity), cellular reactive oxygen species (ROS) production (H2DCFDA intensity) and mitochondrial membrane potential (TMRM intensity) were also assessed in BMDMs. We found that octaparin effectively restored the LPS-induced decline in mitochondrial membrane potential (Fig. 6B). Meanwhile, confocal images showed that octaparin potently inhibited LPS-induced mitochondria-derived superoxide and ROS production (Fig. 6C and D). Furthermore, using a Seahorse extracellular flux analyzer, we measured the oxygen consumption rate (OCR) of BMDMs. LPS stimulation significantly reduced OCR, whereas octaparin partially reversed this decline (Fig. 6E). Quantitative analysis revealed that LPS impaired basal respiration, maximal respiration, and spare respiratory capacity, all of which were significantly improved by octaparin (Fig. 6F). Subsequently, we aimed to determine whether octaparin outperforms heparin and related analogues in enhancing mitochondrial quality and restoring redox homeostasis. We observed that heparin, enoxaparin, and fondaparinux showed little to no ability to reverse LPS-induced mitochondrial membrane potential depolarization, nor could they reduce the production of mitochondria-derived superoxide or intracellular ROS (Fig. 6G, H and I). These findings, which stand in striking contrast to the robust activity of octaparin, provide a basis for understanding why octaparin exerts superior effects to heparin and related analogues in anti-inflammatory efficacy. Collectively, these findings demonstrate that octaparin, in a manner uniquely superior to heparin and related analogues, maintains mitochondrial homeostasis and normalizes mitochondrial metabolic profiles in BMDMs during sepsis.

## 4 Discussion

This study establishes octaparin, a synthetic heparin analogue, as a pioneering therapeutic agent for sepsis by simultaneously targeting mitochondrial integrity and pyroptotic signaling. Our data demonstrate that octaparin fundamentally surpasses conventional heparin through integrated mechanisms that resolve the pathological triad driving sepsis lethality—dysregulated inflammasome activation, bioenergetic collapse, and organelle-directed cell death. Crucially, octaparin achieves this by disrupting the cardiolipin-GSDMD signaling axis while restoring redox homeostasis, positioning it as the first agent bridging anticoagulant safety with mitochondrial repair.

The core mechanistic innovation lies in octaparin's interruption of cardiolipin-dependent pyroptosis execution. Cardiolipin externalization to mitochondrial outer membranes creates binding platforms for the pore-forming GSDMD-N-terminal fragment, an event that permeabilizes organelles and releases mitochondrial DNA as a potent NLRP3 activator [57,58]. Octaparin specifically prevents this cascade by downregulating cardiolipin synthesis and transport genes (*Crls1, Plscr3*), thereby uncoupling pyroptosis from organelle damage. This action is structurally enabled by octaparin's unique high-density sulfation pattern, which may competitively engage cardiolipin or remodel its membrane topology—a mechanism supported by emerging evidence on sulfated glycan-lipid interactions [59,60]. Traditional heparin lack such precision due to heterogeneous sulfation profiles, explaining their inability to mitigate mitochondrial GSDMD translocation.

Inflammasome activation is tightly regulated by upstream signaling networks that detect danger signals and license inflammasome assembly. Canonical inflammasomes are triggered by pathogen-associated molecular patterns (PAMPs) or damage-associated molecular patterns (DAMPs) through receptors such as nucleotide oligomerization domain (NOD)-like receptors, pyrin, or absent in melanoma 2 (AIM2)-like receptors, leading to caspase-1 activation via the adaptor apoptosis-associated speck-like protein containing a CARD (ASC) [61]. Non-canonical inflammasomes are directly activated by cytosolic LPS and endogenous oxidized phospholipids, which bind to caspase-4/5 (human) or caspase-11 (murine) [62]. Octaparin inhibits both pathways, potentially by interfering with upstream priming signals. Priming via TLR4–NF-κB signaling upregulates inflammasome components without directly inducing assembly [[63], [64], [65]]. Octaparin may block LPS-induced TLR4 engagement or downstream NF-κB activation, thereby limiting NLRP3 and pro-cytokine expression. Mitochondrial ROS promote NLRP3 activation and cardiolipin translocation to the outer mitochondrial membrane [55,66], and may enhance GSDMD pore formation. Octaparin suppresses LPS-induced mitochondrial superoxide and ROS production, which may contribute to reduced inflammasome activation.

Concurrently, octaparin orchestrates comprehensive mitochondrial resuscitation. It attenuates superoxide overproduction and cellular reactive oxygen species bursts while repolarizing mitochondrial membranes to restore cristae architecture. Functional recovery is evidenced by significantly enhanced respiratory capacity in inflamed macrophages, indicating restored metabolic flexibility to withstand septic stress. These improvements are amplified by octaparin's reprogramming of macrophage immunometabolism, which suppresses glycolysis-driven inflammasome priming while augmenting bacterial clearance [[67], [68], [69]]. Such reprogramming shifts cytokine profiles toward reparative phenotypes, disrupting the vicious cycle between metabolic dysfunction and inflammation that fuels organ failure [70].

From a translational perspective, octaparin addresses critical gaps in sepsis management. Its synthetic production eliminates batch variability and viral contamination risks inherent to animal-derived heparins, while selective factor Xa inhibition minimizes bleeding complications—a major limitation in coagulopathic sepsis patients [37]. More significantly, octaparin prevents mitochondrial damage in parenchymal cells, directly protecting vital organs from necrosis. This organ-protective efficacy extends beyond acute phase management; by mitigating persistent mitochondrial calcium handling defects in immune cells, octaparin may alleviate the chronic immunosuppression that plagues sepsis survivors [[71], [72], [73]].

Our study has limitations that contextualize these findings. Experimental models, while capturing key hyperinflammatory features, cannot fully replicate human polymicrobial sepsis dynamics or the impact of comorbidities on mitochondrial function [74,75]. Furthermore, although *Crls1* and *Plscr3* downregulation mechanistically explains reduced GSDMD-NT trafficking, direct molecular targets require validation through biophysical approaches such as hydrogen-deuterium exchange mass spectrometry. The effects on mitochondrial fatty acid oxidation and ketogenesis, pathways essential for sepsis recovery, also remain uncharacterized. Future investigations should prioritize three directions. First, validating efficacy in cecal ligation and puncture models with antibiotic stewardship protocols to better mimic clinical practice. Second, dissecting tissue-specific effects using single-cell transcriptomics of Kupffer cells and alveolar macrophages [76]. Third, exploring synergy with immunoadjuvants like interleukin-7 to reverse sepsis-induced lymphopenia [77]. Such studies will clarify whether octaparin's protection extends to aging immune systems or polymicrobial contexts.

In conclusion, octaparin represents a paradigm-shifting therapeutic strategy that chemically defuses the cardiolipin-GSDMD death switch while recalibrating cellular bioenergetics. By concurrently targeting organelle integrity, inflammasome-ROS signaling crosstalk, and immunometabolic paralysis, octaparin establishes a unified therapeutic strategy against sepsis. This approach warrants clinical evaluation in trials that stratify patients using mitochondrial damage biomarkers.

## CRediT authorship contribution statement

**Shule Zhang:** Writing – review & editing, Writing – original draft, Software, Methodology, Investigation, Formal analysis, Data curation, Conceptualization. **Cong Feng:** Software, Project administration, Investigation. **Ning Yu:** Resources, Methodology. **Rui Fang:** Visualization, Validation. **Yingxin Zhang:** Software, Resources. **Simeng Chen:** Data curation. **Lijuan Cao:** Writing – review & editing, Supervision. **Jianfa Zhang:** Writing – review & editing, Supervision, Funding acquisition.

## Declaration of competing interest

The authors declare that they have no known competing financial interests or personal relationships that could have appeared to influence the work reported in this paper.

## Data Availability

All data generated during this study are included in this article. Details of the compounds, primers, and antibodies used for this study are provided in the Supporting Information document.

## Appendix A Supplementary data

Fig. S1 Comparative anti-inflammatory profiling of octaparin, heparin and related analogues. (A) Structural features of heparin, enoxaparin, fondaparinux, and octaparin. (B) ELISA analysis of TNF-α and IL-6 in culture supernatants from THP-1 cells stimulated with LPS (1 μg/mL) in the presence or absence of octaparin (5 μg/mL), heparin (5 μg/mL), enoxaparin (5 μg/mL) and fondaparinux (5 μg/mL) for 24 h. (C) ELISA analysis of TNF-α and IL-6 in culture supernatants from THP-1 cells stimulated with *S. typhimurium* (MOI = 10) in the presence or absence of octaparin (5 μg/mL), heparin (5 μg/mL), enoxaparin (5 μg/mL) and fondaparinux (5 μg/mL) for 24 h. (D) Serum concentrations of IL-1α and IL-1β from mice at 24 h after intraperitoneal injection of LPS (25 mg/kg) or saline. Octaparin (10 or 20 mg/kg) was administered intraperitoneally 30 min after LPS injection. (E) Serum concentrations of IL-1α and IL-1β from mice at 24 h after intraperitoneal injection of *S. typhimurium* (2 × 10^6^ CFU/20 g) or saline. Octaparin (10 or 20 mg/kg) was administered intraperitoneally 2 h after *S. typhimurium* injection. The graphs are shown as individual data points along with mean ± SEM. ∗p < 0.05; ∗∗p < 0.01; ∗∗∗p < 0.001. Statistical analyses by one-way ANOVA test. Fig. S1

Fig. S2 Flow cytometry confirms THP-1 differentiation into macrophages via CD68 upregulation. THP-1 cells were stimulated with 20 ng/mL phorbol 12-myristate 13-acetate (PMA; Sigma-Aldrich) or vehicle for 48 h. (A) The differentiation of THP-1 cells into macrophages was confirmed by FACS analysis using PE-conjugated anti-human CD68 antibody. (B) The representative flow cytometry histograms were presented as cell counts over the log fluorescence intensity of PE-conjugated anti-human CD68, whereas quantification of PE-CD68 expression was presented as mean fluorescence intensity (MFI). The graphs are shown as individual data points along with mean ± SEM. ∗p < 0.05; ∗∗p < 0.01; ∗∗∗p < 0.001. Statistical analyses were performed by Student's t-test. Fig. S2

Fig. S3 Octaparin reduces serum AST and BUN levels in *S. typhimurium*-infected septic mice. Mice received intraperitoneal injections of *S. typhimurium* (2 × 10^6^ CFU/20 g) or sterile saline. Octaparin (20 mg/kg) was administered intraperitoneally 2 h following *S. typhimurium* challenge. Mice were sacrificed 48 h later for analyses. (A-B) Serum levels of AST (A) and BUN (B). The graphs are shown as individual data points along with mean ± SEM. ∗p < 0.05; ∗∗p < 0.01; ∗∗∗p < 0.001. Statistical analyses by one-way ANOVA test. Fig. S3

Fig. S4 Octaparin boosts BMDM phagocytic capacity. (A) The relative phagocytic activity of BMDMs stimulated with indicated doses of octaparin for 24 h was assessed via neutral red uptake. (B) BMDMs were first incubated with *S. typhimurium-GFP* at a MOI of 10 for 1 h, followed by stimulation with the indicated doses of octaparin for an additional 24 h prior to phagocytic activity quantification. (C) BMDMs were first incubated with *S. typhimurium-GFP* at a MOI of 10 for 1 h, followed by stimulation with the indicated doses of octaparin for an additional 6 h prior to quantification of phagocytic activity. Representative images are shown. Scale bars, 20 μm. (D) Bacterial clearance capacity was evaluated using data from (C) by calculating the percentage decrease in extracellular fluorescence. (E) BMDMs were first incubated with *S. typhimurium-GFP* at a MOI of 5 for 1 h, followed by stimulation with the indicated doses of octaparin for an additional 24 h. Representative confocal microscopy images show PI^+^ GFP^+^ cells. Scale bar, 20 μm. (F) The percentage of PI^+^ GFP^+^ double-positive cells in (E). The graphs are shown as individual data points along with mean ± SEM. ∗p < 0.05; ∗∗p < 0.01; ∗∗∗p < 0.001. Statistical analyses by one-way ANOVA test. Fig. S4

Fig. S5 Octaparin suppresses canonical and non-canonical inflammasome activation in BMDMs. (A) Cell viability, LDH release, and IL-1β secretion in BMDMs primed with LPS (1 μg/mL) for 2 h, followed by nigericin (10 μM) stimulation for 2 h in the presence or absence of indicated octaparin doses. (B) Cell viability, LDH release, and IL-1β secretion in BMDMs treated with LPS (1 μg/mL) + HMGB1 (100 ng/mL) for 24 h in the presence or absence of indicated octaparin doses. The graphs are shown as individual data points along with mean ± SEM. ∗p < 0.05; ∗∗p < 0.01; ∗∗∗p < 0.001. Statistical analyses by one-way ANOVA test. Fig. S5
