# Supplementary material for: Synthetic anticoagulant octaparin targets mitochondrial cardiolipin-GSDMD axis to rescue redox homeostasis in sepsis
Source: Redox Biol. 2025 Sep 22;87:103877. doi: 10.1016/j.redox.2025.103877 (PMC12495058; doi:10.1016/j.redox.2025.103877)

Supplemental Figure 1. Comparative anti-inflammatory profiling of octaparin, heparin and its analogues.

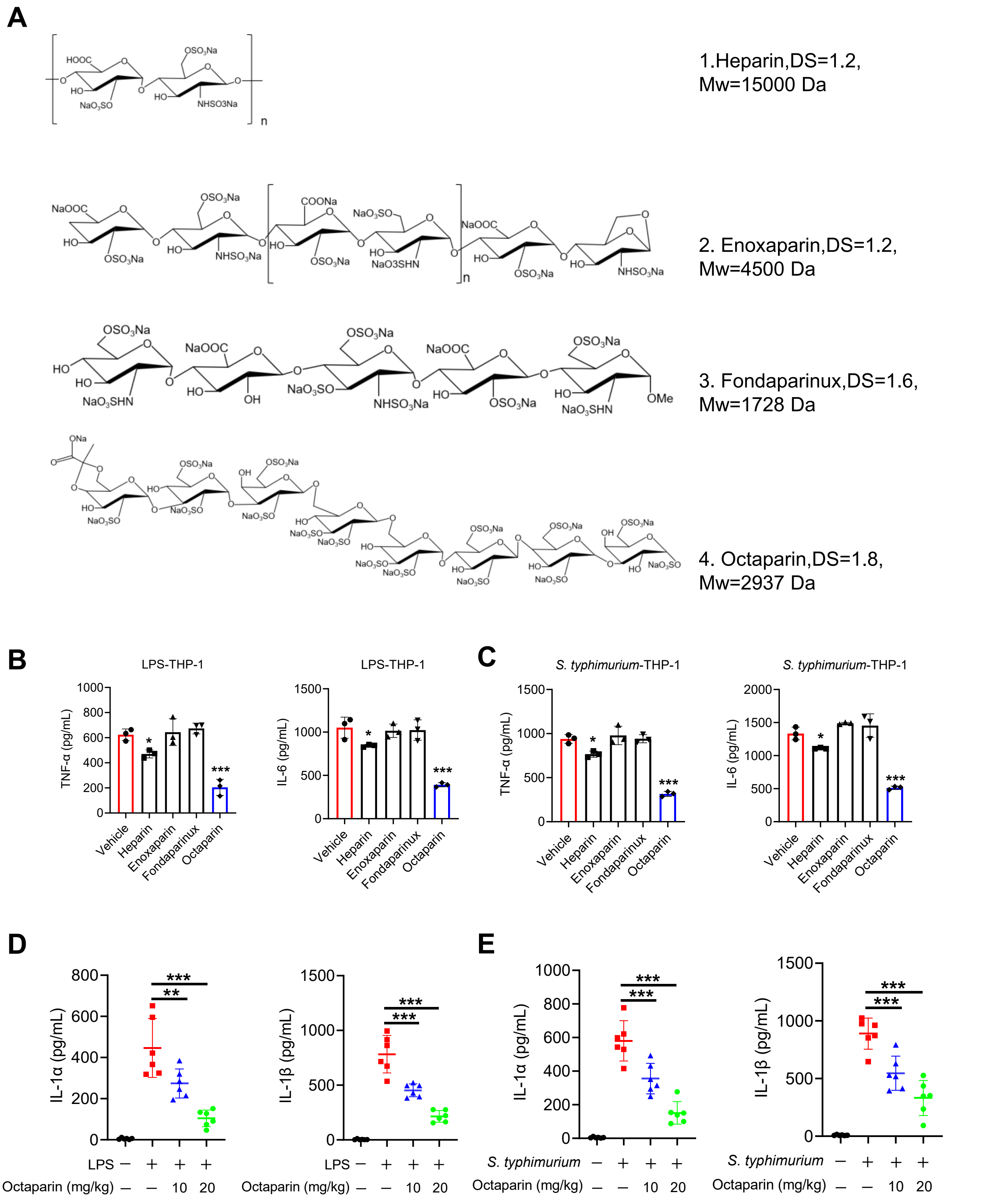

Supplement: Fig. S1 — Comparative anti-inflammatory profiling of octaparin, heparin and related analogues. (A) Structural features of heparin, enoxaparin, fondaparinux, and octaparin. (B) ELISA analysis of TNF-α and IL-6 in culture supernatants from THP-1 cells stimulated with LPS (1 μg/mL) in the presence or absence of octaparin (5 μg/mL), heparin (5 μg/mL), enoxaparin (5 μg/mL) and fondaparinux (5 μg/mL) for 24 h. (C) ELISA analysis of TNF-α and IL-6 in culture supernatants from THP-1 cells stimulated with S. typhimurium (MOI = 10) in the presence or absence of octaparin (5 μg/mL), heparin (5 μg/mL), enoxaparin (5 μg/mL) and fondaparinux (5 μg/mL) for 24 h. (D) Serum concentrations of IL-1α and IL-1β from mice at 24 h after intraperitoneal injection of LPS (25 mg/kg) or saline. Octaparin (10 or 20 mg/kg) was administered intraperitoneally 30 min after LPS injection. (E) Serum concentrations of IL-1α and IL-1β from mice at 24 h after intraperitoneal injection of S. typhimurium (2 × 10^6^ CFU/20 g) or saline. Octaparin (10 or 20 mg/kg) was administered intraperitoneally 2 h after S. typhimurium injection. The graphs are shown as individual data points along with mean ± SEM. ∗p < 0.05; ∗∗p < 0.01; ∗∗∗p < 0.001. Statistical analyses by one-way ANOVA test. [file mmc1.pdf]
