# Supplementary material for: Synthetic anticoagulant octaparin targets mitochondrial cardiolipin-GSDMD axis to rescue redox homeostasis in sepsis
Source: Redox Biol. 2025 Sep 22;87:103877. doi: 10.1016/j.redox.2025.103877 (PMC12495058; doi:10.1016/j.redox.2025.103877)

Supplemental Figure 2. Flow cytometry confirms THP-1 differentiation into macrophages via CD68 upregulation.

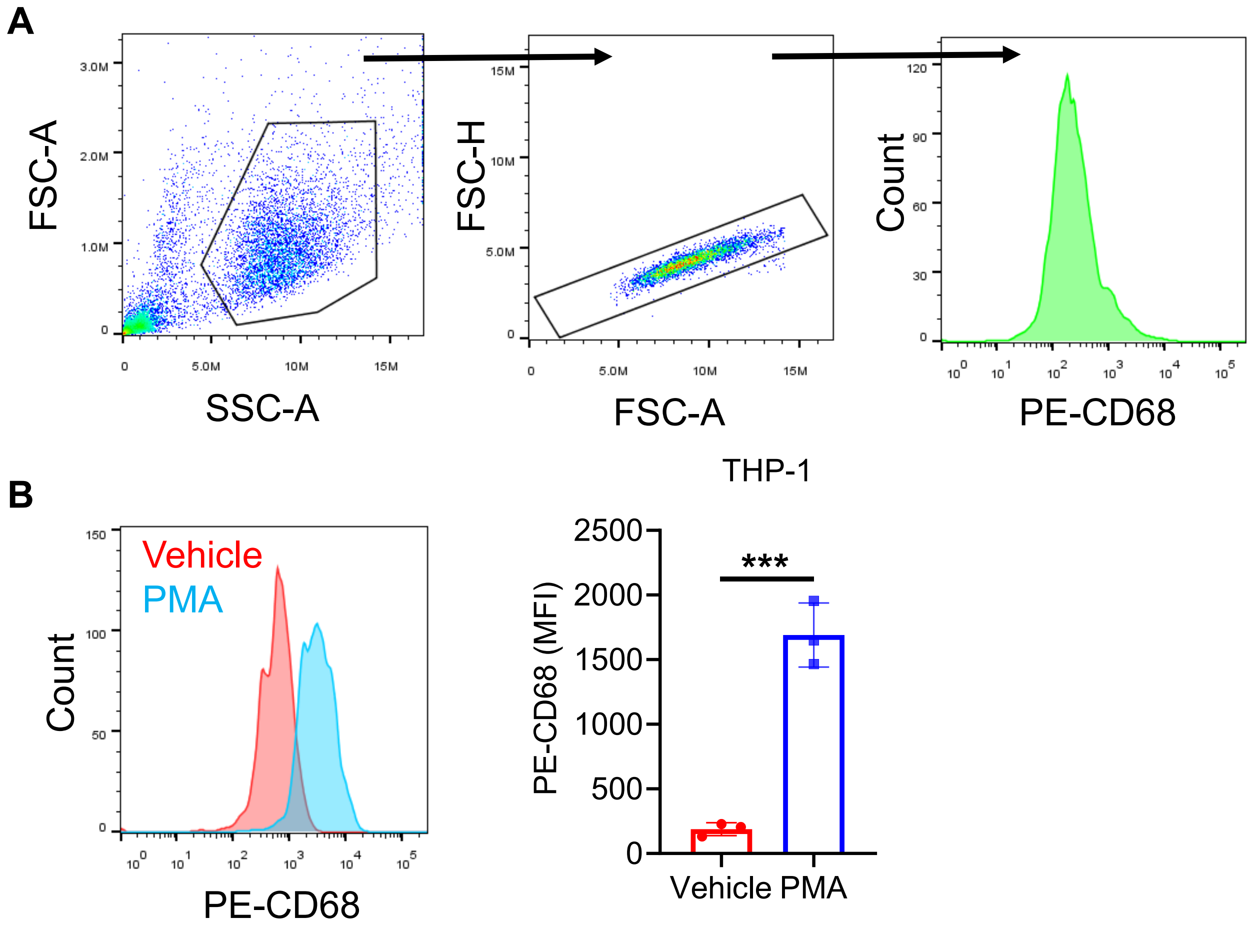

Supplement: Fig. S2 — Flow cytometry confirms THP-1 differentiation into macrophages via CD68 upregulation. THP-1 cells were stimulated with 20 ng/mL phorbol 12-myristate 13-acetate (PMA; Sigma-Aldrich) or vehicle for 48 h. (A) The differentiation of THP-1 cells into macrophages was confirmed by FACS analysis using PE-conjugated anti-human CD68 antibody. (B) The representative flow cytometry histograms were presented as cell counts over the log fluorescence intensity of PE-conjugated anti-human CD68, whereas quantification of PE-CD68 expression was presented as mean fluorescence intensity (MFI). The graphs are shown as individual data points along with mean ± SEM. ∗p < 0.05; ∗∗p < 0.01; ∗∗∗p < 0.001. Statistical analyses were performed by Student's t-test. [file mmc2.pdf]
