# Supplementary material for: Synthetic anticoagulant octaparin targets mitochondrial cardiolipin-GSDMD axis to rescue redox homeostasis in sepsis
Source: Redox Biol. 2025 Sep 22;87:103877. doi: 10.1016/j.redox.2025.103877 (PMC12495058; doi:10.1016/j.redox.2025.103877)

Supplemental Figure 3. Octaparin reduces serum AST and BUN levels in *S. typhimurium*-infected septic mice.

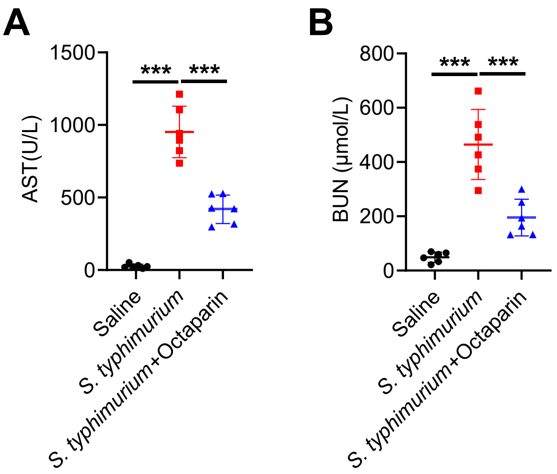

Supplement: Fig. S3 — Octaparin reduces serum AST and BUN levels in S. typhimurium-infected septic mice. Mice received intraperitoneal injections of S. typhimurium (2 × 10^6^ CFU/20 g) or sterile saline. Octaparin (20 mg/kg) was administered intraperitoneally 2 h following S. typhimurium challenge. Mice were sacrificed 48 h later for analyses. (A-B) Serum levels of AST (A) and BUN (B). The graphs are shown as individual data points along with mean ± SEM. ∗p < 0.05; ∗∗p < 0.01; ∗∗∗p < 0.001. Statistical analyses by one-way ANOVA test. [file mmc3.pdf]
