# Supplementary figures and images for: Synthetic anticoagulant octaparin targets mitochondrial cardiolipin-GSDMD axis to rescue redox homeostasis in sepsis
Source: Redox Biol. 2025 Sep 22;87:103877. doi: 10.1016/j.redox.2025.103877 (PMC12495058; doi:10.1016/j.redox.2025.103877)

Supplemental Figure 4. Octaparin boosts BMDM phagocytic capacity.

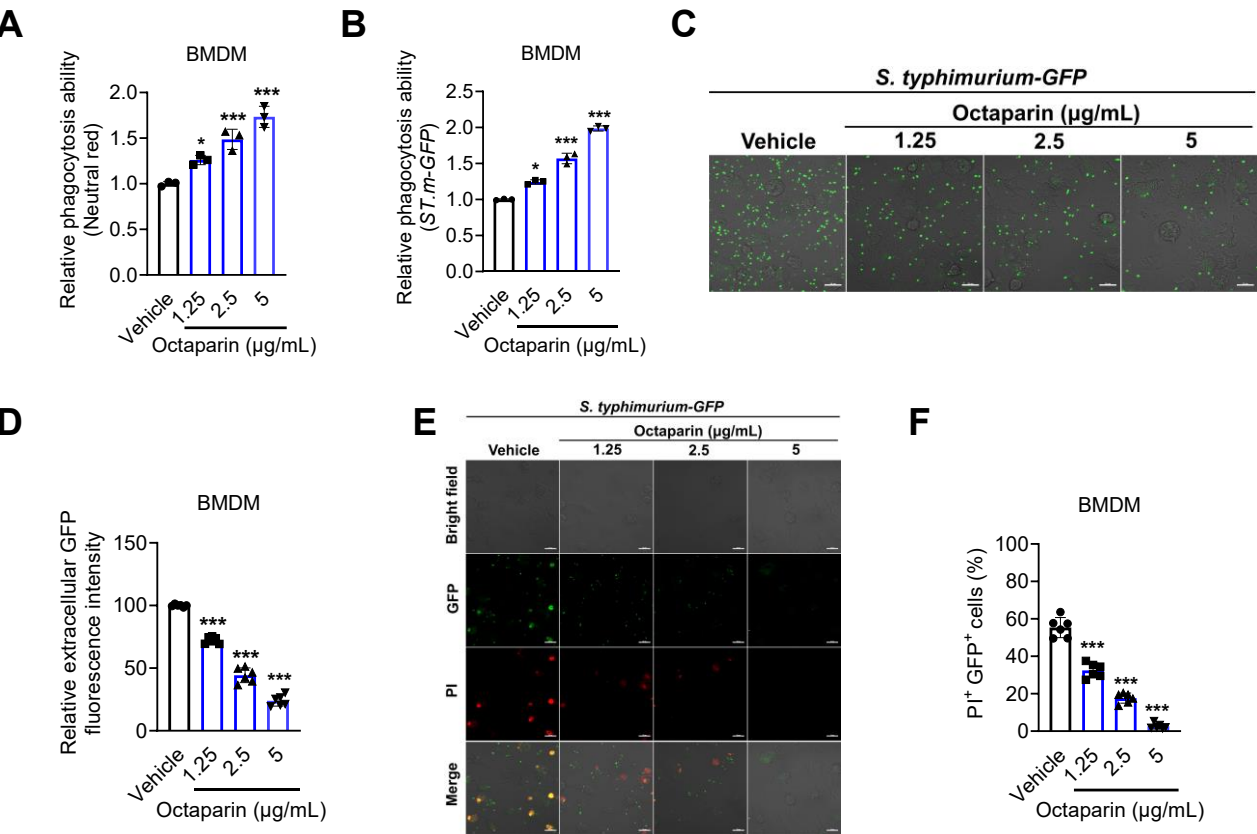

Supplement: Fig. S4 — Octaparin boosts BMDM phagocytic capacity. (A) The relative phagocytic activity of BMDMs stimulated with indicated doses of octaparin for 24 h was assessed via neutral red uptake. (B) BMDMs were first incubated with S. typhimurium-GFP at a MOI of 10 for 1 h, followed by stimulation with the indicated doses of octaparin for an additional 24 h prior to phagocytic activity quantification. (C) BMDMs were first incubated with S. typhimurium-GFP at a MOI of 10 for 1 h, followed by stimulation with the indicated doses of octaparin for an additional 6 h prior to quantification of phagocytic activity. Representative images are shown. Scale bars, 20 μm. (D) Bacterial clearance capacity was evaluated using data from (C) by calculating the percentage decrease in extracellular fluorescence. (E) BMDMs were first incubated with S. typhimurium-GFP at a MOI of 5 for 1 h, followed by stimulation with the indicated doses of octaparin for an additional 24 h. Representative confocal microscopy images show PI+ GFP+ cells. Scale bar, 20 μm. (F) The percentage of PI+ GFP+ double-positive cells in (E). The graphs are shown as individual data points along with mean ± SEM. ∗p < 0.05; ∗∗p < 0.01; ∗∗∗p < 0.001. Statistical analyses by one-way ANOVA test. [file mmc4.pdf]
