# Supplementary material for: Synthetic anticoagulant octaparin targets mitochondrial cardiolipin-GSDMD axis to rescue redox homeostasis in sepsis
Source: Redox Biol. 2025 Sep 22;87:103877. doi: 10.1016/j.redox.2025.103877 (PMC12495058; doi:10.1016/j.redox.2025.103877)

Supplemental Figure 5. Octaparin suppresses canonical and non-canonical inflammasome activation in BMDMs.

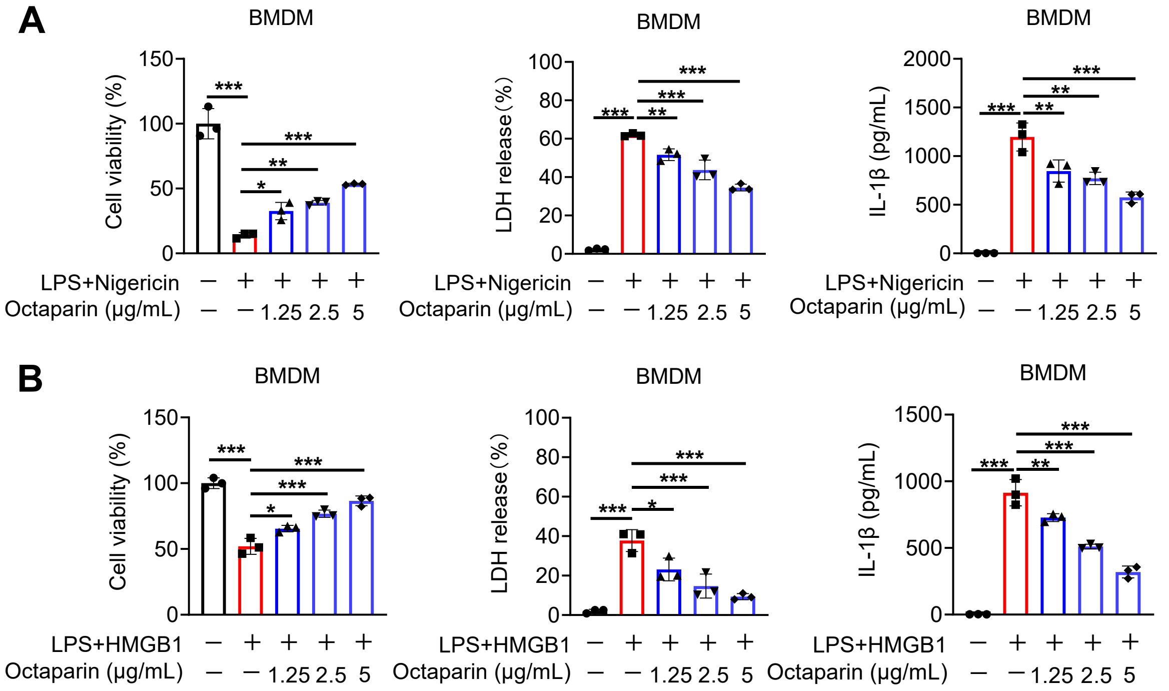

Supplement: Fig. S5 — Octaparin suppresses canonical and non-canonical inflammasome activation in BMDMs. (A) Cell viability, LDH release, and IL-1β secretion in BMDMs primed with LPS (1 μg/mL) for 2 h, followed by nigericin (10 μM) stimulation for 2 h in the presence or absence of indicated octaparin doses. (B) Cell viability, LDH release, and IL-1β secretion in BMDMs treated with LPS (1 μg/mL) + HMGB1 (100 ng/mL) for 24 h in the presence or absence of indicated octaparin doses. The graphs are shown as individual data points along with mean ± SEM. ∗p < 0.05; ∗∗p < 0.01; ∗∗∗p < 0.001. Statistical analyses by one-way ANOVA test. [file mmc5.pdf]
